# BEYOND BINGO: THE IMPACT OF ACTIVITY ENGAGEMENT IN AN ALL-AFRICAN AMERICAN ASSISTED LIVING COMMUNITY

**DOI:** 10.1093/geroni/igz038.3480

**Published:** 2019-11-08

**Authors:** Inneke L Vargas, Alexis A Bender, Candace L Kemp, Molly M Perkins

**Affiliations:** 1 Wichita State University, Wichita, Kansas, United States; 2 Emory University, Atlanta, Georgia, United States; 3 Georgia State University, Atlanta, Georgia, United States; 4 Division of General Medicine and Geriatrics, Emory University School of Medicine, Atlanta, Georgia, United States

## Abstract

More than one million older adults reside in assisted living (AL) communities in America. This figure is projected to double by the year 2030. It is typical for residents in these communities to have cognitive and physical impairments requiring differing levels of care. Due in part to these impairments, it is vital to the health and well-being of residents to participate in meaningful recreational activities. This secondary analysis of ethnographic observations totaling 818 hours and semi-structured interviews with 25 residents enrolled in an NIA-funded study (5R01AG047408) explores in depth barriers and facilitators to activity engagement in a large (90+ bed) moderate-income, all-African-American AL community located in a large urban city in the southern US. Residents range in age from 59 to 103 (mean = 85) and are predominantly female. We linked our findings from thematic analysis to six domains of quality palliative care identified by the 2018 National Consensus Project (NCP) Guidelines for Quality Palliative Care: cultural, physical, psychological, social, structures and processes, and spiritual aspects of care. Key barriers include limitations related to staffing (a low staff-to-resident ratio and high staff turnover) and activities that do not adequately address needs of residents with varying interests and abilities. A robust daily devotion and other activities that incorporate culturally relevant music are activities highly valued by most residents. Implications of these findings contribute to a larger effort to create positive change in the structures and processes of care in AL and can inform best practices for palliative care within these communities.

